# A New Citrinin Derivative from the Indonesian Marine Sponge-Associated Fungus *Penicillium citrinum*

**DOI:** 10.3390/md18040227

**Published:** 2020-04-24

**Authors:** Aninditia Sabdaningsih, Yang Liu, Ute Mettal, John Heep, Riyanti, Lei Wang, Olvi Cristianawati, Handung Nuryadi, Mada Triandala Sibero, Michael Marner, Ocky Karna Radjasa, Agus Sabdono, Agus Trianto, Till F. Schäberle

**Affiliations:** 1 Department of Coastal Resources Management, Faculty of Fisheries and Marine Sciences, Diponegoro University, Semarang 50275, Indonesia; olvi.cristiana@yahoo.com; 2 Department of Aquatic Resources, Faculty of Fisheries and Marine Sciences, Diponegoro University, Semarang 50275, Indonesia; 3 Institute for Insect Biotechnology, Justus-Liebig-University of Giessen, 35392 Giessen, Germany; Ute.Mettal@chemie.uni-giessen.de (U.M.); or riyanti.anti@gmail.com (R.); Lei.Wang@agrar.uni-giessen.de (L.W.); 4 Tropical Marine Biotechnology Laboratory, Diponegoro University, Semarang 50275, Indonesia; handung.nuryadi87@gmail.com (H.N.); madatriandala@hotmail.com (M.T.S.); ocky_radjasa@yahoo.com (O.K.R.); agus_sabdono@yahoo.com (A.S.); 5 Marine Natural Product Laboratory, Diponegoro University, Semarang 50275, Indonesia; agustrianto.undip@gmail.com; 6 Department of Bioresources of the Fraunhofer Institute for Molecular Biology and Applied Ecology (IME), 35392 Giessen, Germany; John.Heep@ime.fraunhofer.de (J.H.); Michael.Marner@ime.fraunhofer.de (M.M.); 7 Faculty of Fisheries and Marine Science, Jenderal Soedirman University, Purwokerto 53122, Indonesia; 8 Graduate School of Engineering and Science, University of the Ryukyus, 1 Senbaru, Nihihara, Okinawa 903-0213, Japan; 9 Department of Marine Sciences, Faculty of Fisheries and Marine Sciences, Diponegoro University, Semarang 50275, Indonesia; 10 Ministry of Research and Technology of the Republic of Indonesia, Jakarta 10340, Indonesia; 11 German Center for Infection Research (DZIF), Partner Site Giessen-Marburg-Langen, 35392 Giessen, Germany

**Keywords:** antibacterial, citrinin derivatives, marine-derived fungi, *Penicillium citrinum*, penicitrinone, marine sponges

## Abstract

Sponge-associated fungi are attractive targets for the isolation of bioactive natural products with different pharmaceutical purposes. In this investigation, 20 fungi were isolated from 10 different sponge specimens. One isolate, the fungus *Penicillium citrinum* strain WK-P9, showed activity against *Bacillus subtilis* JH642 when cultivated in malt extract medium. One new and three known citrinin derivatives were isolated from the extract of this fungus. The structures were elucidated by 1D and 2D NMR spectroscopy, as well as LC-HRMS. Their antibacterial activity against a set of common human pathogenic bacteria and fungi was tested. Compound **2** showed moderate activity against *Mycobacterium smegmatis* ATCC607 with a minimum inhibitory concentration (MIC) of 32 µg/mL. Compound **4** exhibited moderate growth inhibition against *Bacillus subtilis* JH642, *B. megaterium* DSM32, and *M. smegmatis* ATCC607 with MICs of 16, 16, and 32 µg/mL, respectively. Furthermore, weak activities of 64 µg/mL against *B. subtilis* DSM10 and *S. aureus* ATCC25923 were observed for compound **4**.

## 1. Introduction

The discovery of bioactive compounds from the marine environment has increased over the last two decades, showing the potential of this habitat for the discovery of novel structures [1,2]. As an example, marine sponges are a reported source for agents with diverse biological activities, e.g., antimicrobial or cytostatic activity [3,4,5,6,7,8]. However, in order to conduct preclinical and clinical trials and to develop a promising hit compound into a lead candidate for a marketed drug, continuous substance supply is crucial. For instance, one kilogram wet weight of the sponge *Lissodendoryx* sp. yielded only 400 µg of halichondrin B [9]. Only chemical synthesis of the complex natural product enabled further development as a lead structure, which finally led to the synthesis of the structurally simplified and pharmaceutically optimized analog eribulin [10]. Furthermore, sponges have an important role in their ecosystems [11]. Therefore, exploitation of this bioresource must be avoided. Hence, for compounds that are produced by sponge-associated microorganisms, e.g., fungi, it would be desirable to cultivate the latter. In that way, the supply issue should be easier to solve, since fermentation can be done in a more sustainable fashion under laboratory conditions rather than harvesting the slow-growing macroorganisms. Such fungi were shown to produce a wide range of chemically diverse compounds, displaying a variety of biological activities, e.g., antibacterial, anti-inflammatory, antiviral, and anticancer activity [12,13,14,15,16,17]. The antibacterial compounds aspochalasin B and D were successfully isolated from a crude extract of *Aspergillus flavipes.* This fungus was associated with a sponge of the *Demospongiae* class, and the fungal extract exhibited growth inhibition of *Bacillus subtilis, Staphylococcus aureus* and Methicillin-resistant *S. aureus* (MRSA) [18]. Moreover, the fungus *Neosartorya fennelliae* KUFA 0811, associated with the marine sponge *Clathria reinwardtii* and a producer of the antibacterial agents paecilin E and dankastetrone A. Paecilin E, showed activity against *S. aureus* ATCC 29213 and *Enterobacter faecalis* ATCC 29212, while dankastetrone A was active against *E. faecalis* ATCC 29212 and the multidrug-resistant VRE *Enterobacter faecalis* A5/102 [19]. Thus, compounds from sponge-associated fungi already proved to be highly valuable for the detection of interesting biological and pharmaceutical activities and it is expected that many more new natural products are waiting to be discovered.

Considering promising bioresources, one valid approach is to select species originating from challenging environments, e.g., habitats with a high biodiversity. The coral triangle which has rich biodiversity and the greatest repository of marine life, represents such an environment [20]. Therefore, we report herein, the sponge collection from the Wakatobi National Park, located in South East Sulawesi, Indonesia. Associated fungi were retrieved, molecules were isolated based on bioactivity, their structures elucidated, and their antibacterial activities tested.

## 2. Results

### 2.1. Sample Collection, Isolation, and Structure Elucidation

**Figure 1:**
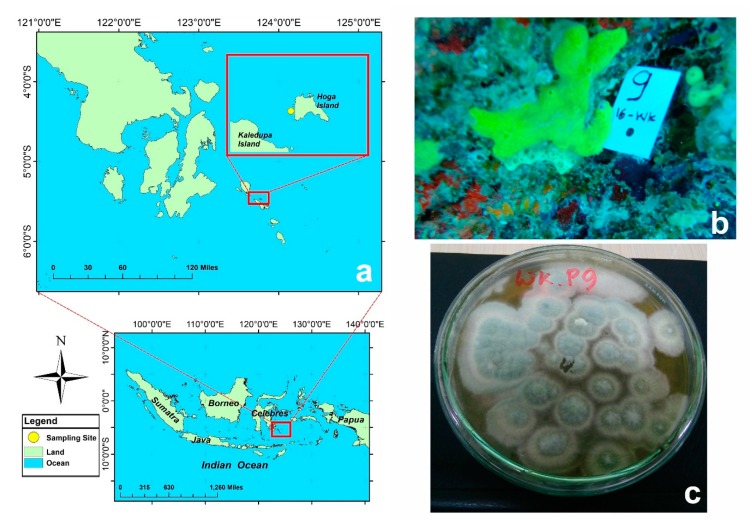
**a:** Sampling site at Wakatobi National Park; **b:** underwater picture of sponge *Suberea* sp. WK-P9; **c:** picture of the therefrom isolated fungus *Penicillium citrinum* WK-P9, grown on a malt extract agar plate.

**Figure 2:**
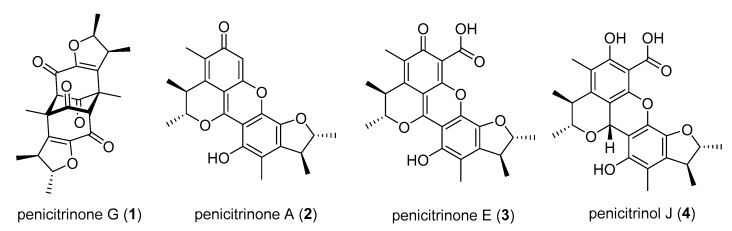
Structures of the isolated compounds from sponge-associated fungus *P. citrinum* WK-P9.

During our ongoing research to discover antibacterial compounds, 10 sponges were collected from Hoga Island located in Wakatobi National Park (Figure 1). This region was classified as a Marine Protected Area in 2002 [21,22]. Between 1993 and 2013, 112 marine natural products have been isolated from various organisms, and at least 51 compounds have been reported as a pharmaceutical agent [23]. From our 10 sponge samples, 20 fungal colonies were isolated, mainly belonging to the genera *Aspergillus* and *Penicillium*. Initial activity screenings of those 20 fungi were performed and the crude extract of *Penicillium citrinum* WK-P9, isolated from a *Suberea* sp., showed antibacterial activity against *Bacillus subtilis* and *Bacillus megaterium*, which prompted us to isolate the corresponding specialized metabolites. The ethyl acetate (EtOAc) extract of the sponge-derived fungus *P. citrinum* WK-P9 cultivated in malt extract broth medium was fractionated by medium pressure liquid chromatography (MPLC) with reverse phase silica gel C18. The active fractions obtained by bioassay-guided fractionation were further purified by size exclusion chromatography and by HPLC. This procedure led to the isolation of one new citrinin derivative (**1**), and 3 known compounds, namely, penicitrinone A (**2**), penicitrinone E (**3**), penicitrinol J (**4**) (Figure 2).

Compound **1** was isolated as a white amorphous solid with an optical rotation value of ${\left[\alpha \right]}_{D}^{24}=$ 16.25 (*c* 0.03, MeOH). Its molecular formula was established as C_22_H_24_O_6_, based on the prominent pseudomolecular ion peaks at *m/z* 385.1653 [M + H]^+^ and 407.1489 [M + Na]^+^ in the LC-HRESIMS spectrum (Figure S7). The ^1^H NMR spectrum of **1** revealed two oxymethine protons (H-1, δ 4.43; H-1′, δ 4.37), four methine protons (H-6′, δ 3.71; H-6, δ 3.63; H-2, δ 2.99; H-2′, δ 2.91), and six methyl groups (H-10′, δ 1.36; H-11′, δ 1.332; H-11, δ 1.325; H-9′, δ 1.30; H-9, δ 1.24; H-10, δ 1.22) (Figure S1). The ^13^C NMR spectra suggested four carbonyl groups (C-5, δ 199.5; C-5′, δ 199.0; C-7, δ 184.7; C-7′, δ 184.2), two oxygenated *sp*^2^ carbons (C-8, δ 150.3; C-8′, δ 150.8), two *sp*^2^ carbons (C-3, δ 140.0; C-3′, δ 139.2), four *sp*^3^ carbons in proximity to an electron withdrawing group (C-1, δ 89.2; C-1′, δ 88.3; C-6, δ 73.1; C-6′, δ 71.9), another four *sp*^3^ carbons (C-4, δ 55.6; C-4′, δ 55.1; C-2′, δ 47.1, C-2, δ 45.3), and six methyl groups (C-9′, δ 20.7; C-9, δ 20.6; C-10, δ 20.3; C-10′, δ 19.3; C-11, δ 16.1; C-11′, δ 14.5) (Figure S2). Further analysis of the 1D and 2D NMR data, allowed for the assignment of two identical planar moieties. The unequivocal assignments of the ^1^H and ^13^C NMR data for each unit were based on the analysis of the COSY, heteronuclear single quantum correlation (HSQC), and heteronuclear multiple bond correlation (HMBC) spectra (Table 1, Figures S3–S5). 

In the COSY spectrum, two spin systems are observed. One of them consists of H-9, H-1, H-2, and H-10, while the other one comprises H-9′, H-1′, H-2′, and H-10′. This is also confirmed by HMBC correlations. For the first molecular fragment, the HMBC correlations range from H-1 to C-3 and C-8, from H-2 to C-3, C-8, and C-10, from H-9 to C-1, C-2, and from H-10 to C-1, C-2 and C-3. Correspondingly, the second fragment shows HMBC correlations from H-1′ to C-3′ and C-8′, from H-2′ to C-3′, C-8′, and C-10′, from H-9′ to C-1′, C-2′, and from H-10′ to C-1′, C-2′ and C-3′.

**Figure 3:**
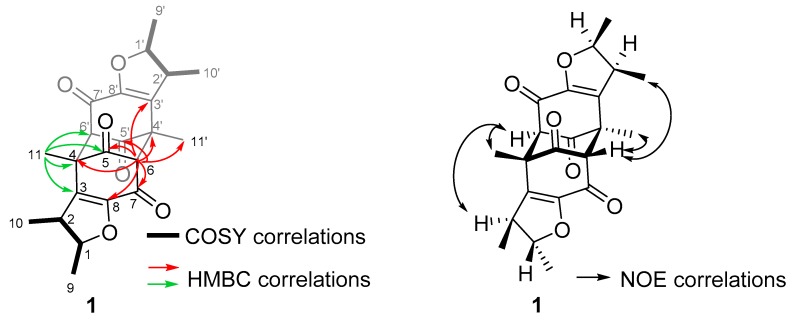
Key HMBC and NOE correlations of compound **1**.

Further HMBC correlations from H-6 to C-5, C-7, and C-8, as well as the correlations from H-11 to C-3, C-4, C-5, and C-8 established the first monomeric unit as shown in Figure 3 (black). Accordingly, the second monomeric unit (Figure 3, grey) is confirmed by HMBC correlations from H-6′ to C-5′, C-7′, and C-8′, as well as the correlations from H-11′ to C-3′, C-4′, C-5′, and C-8′.

As can be concluded from the HMBC correlations from H-11 to C-6′, from H-6 to C-3′, C-4′ and C-11′, from H-11′ to C-6, and from H-6′ to C-3, C-4 and C-11, the two monomeric units are connected via bonds between C-4 and C-6′, as well as C-6 and C-4′. Therefore, compound **1** was elucidated as a new natural product, for which we propose the name penicitrinone G.

The relative configuration was determined based on NOE analysis together with the coupling constant. The correlations from H-6 to H-11′, and from H-6′ to H-11 indicated that both H-6 and H-6′, as well as H-11 H-11′ are in equatorial position, which established the central ring as chair configuration. The relative configuration of both dihydrofuran rings was determined mainly by comparing the coupling constant with the reported compounds and simulated ^1^H NMR spectra of *trans* and *cis* stereoisomers [24]. The stereochemistry of H-1 and H-2 was determined as *trans* configuration. This assignment was based on the coupling constant of compound **1** with *J*_1,2_ = 3.6 Hz, which is close to the reported penicitrinone E (*J*_2′,3′_ = 4.3 Hz) as well as the simulated ^1^H NMR spectrum (*J* = 4.0 Hz) [24]. However, the relative configuration of H-1′ and H-2′ was elucidated to be *cis* due to the coupling constant of *J*_1′,2′_ = 6.4 Hz, which was more similar to the coupling constant for the simulated *cis* configuration (*J* = 8.2 Hz) [24]. The different configurations of those two dihydrofuran rings can also be confirmed by the NOE correlations. For the first dihydrofuran ring NOE correlations range from both H-11 and H-6′ to H-2, while for the other dihydrofuran ring, there is a NOE correlation from H-6 to H-10′ instead of H-2. Therefore, the relative configuration of compound **1** was assigned as shown in Figure 3.

For compounds **2**–**4**, structure elucidation based on 1D and 2D NMR spectra as well as LC-HRMS, led to the known secondary metabolites penicitrinone A (**2**) [25], penicitrinone E (**3**) [26], and penicitrinol J (**4**) [26]. Those structures were additionally confirmed by comparison with the published NMR data [25,26].

### 2.2. Bioactivity

In order to gain insight into the biological activity of compounds **1**–**4**, they were tested against a panel of eight different bacteria (*Bacillus megaterium* DSM32, *Bacillus subtilis* JH642, *Bacillus subtilis* DSM10, *Micrococcus luteus* ATCC 4698, *Mycobacterium smegmatis* ATCC607, *Listeria monocytogenes* DSM20600, *Staphylococcus aureus* ATCC25923, *Escherichia coli* K12), as well as against the yeast *Candida albicans* FH2173, and the mold fungus *Aspergillus flavus* ATCC9170. The results of the bioactivity assays are summarized in Table 2. Only compounds **2** and **4** revealed moderate activities against Gram-positive strains, while no effect was observed against Gram-negative bacteria and fungi.

## 3. Discussion 

### 3.1. Bioactivity

As can be seen in Table 2, penicitrinol J (**4**) exhibited moderate activity against *B. megaterium*, *B. subtilis* JH642, and *M. smegmatis* (with corresponding MICs of 16, 16, and 32 µg/mL), whereas penicitrinone A (**2**) only showed weak activity against *M. smegmatis*. The activity against *M. smegmatis* was of the same magnitude for compounds **2** and **4** (MIC of 32 µg/mL, respectively). On the other hand, penicitrinone E (**3**) and the new compound **1** were inactive against the chosen test strains. Comparing the structures of compounds **2** and **3**, the only difference is an additional carboxylic acid group in compound **3**, which might imply that the carboxylic acid group slightly decreases the antibacterial activity. A likewise structural comparison of compounds **3** and **4** in relation to their bioactivities, suggests that phenolic systems (**4**) provide increased antibacterial potency in contrast to chinoid systems (**3**). In this case, the benefit of the aromatic system even seems to outweigh the effect of the carboxylic acid function. The increased activity of penicitrinol J (**4**) as opposed to penicitrinone E (**3**) was previously observed in a paper diffusion assay, but not quantified by determination of the respective MIC values [26]. The idea, that the antibacterial activity might be related to the presence of a phenolic system can be supported by the results of Yang et al. [27], who reported that penicitrinol A shows superior antibiotic activity to penicitrinone A (**2**) when tested against *M. luteus* and *E. coli*. Whether the difference in bioactivity of compounds **3** and **4** is to be attributed to structural factors or to their different redox properties still needs to be investigated. For the related compound citrinin, its redox properties seem to play an important role for its bioactivity, especially for its reported anti-fungal effect [28,29,30]. In contrast to previous reports, in the present study no activity could be detected for penicitrinone A (**2**) against *M. luteus* [27], *E. coli* [27] or *B. megaterium* [31].The lack of anti-fungal activity determined for compound **2** against *C. albicans* and *A. flavus* on the other hand, is in accordance with earlier findings [25].

### 3.2. Biosynthesis

**Figure 4:**
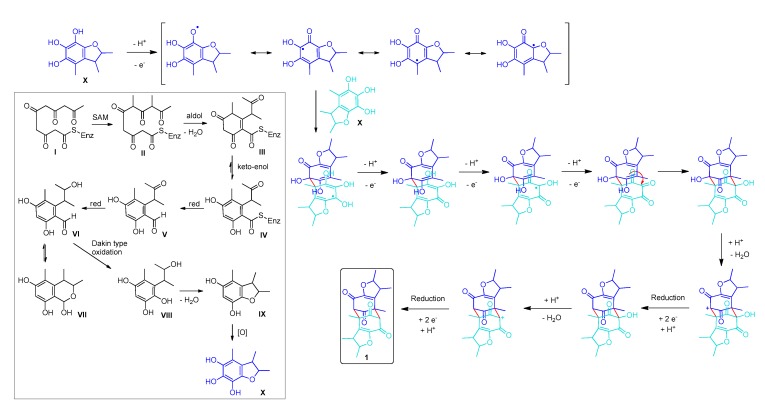
Proposed biosynthetic pathway of compound **1**.

Due to the interesting structure of the new compound **1**, we were wondering what type of reactions are involved in its biosynthesis. To hypothesize a biosynthetic pathway for compound **1**, first the proposed biosyntheses of penicitrinone and penicitrinol derivatives were analyzed [26,32,33,34]. In contrast to the biosynthesis of most citrinin derivatives, which include a Diels–Alder reaction, we propose a radical pathway for the formation of compound **1**. This assumption is based on a similar mechanism reported for the formation of the structurally related compound dibefurin [35] (Figure 4). We assume that the key precursor for the formation of compound **1** is 2,3,4-trimethyl-5,6,7-trihydroxy-2,3-dihydrobenzofuran. Even though this substance itself is not known to date, it constitutes one of the monomers forming penicitol B [36]. The key intermediate might arise from the oxidation of 2,3,4-trimethyl-5,7-dihydroxy-2,3-dihydrobenzofuran, a compound frequently found in extracts of *P. citrinum* species [25,26,37]. This substance in turn, could either be produced directly via the polyketide pathway, as shown in Figure 4, or might alternatively be a decomposition product of citrinin as proposed by Clark et al. [24]. Starting point of our hypothesis is the pentaketide **I**, which is *C*-methylated by a radical SAM enzyme and then yields after aldol condensation and keto-enol tautomerization the enzyme bound aromatic compound **IV**. Labelling experiments for the elucidation of the citrinin biosynthesis [33] seem to support our assumption of *C*-methylation of an unbranched pentaketide. Reductive release from the enzyme [33], yielding the aromatic aldehyde **V** is also suggested in analogy to the biosynthesis of citrinin. After reduction of the ketone to the alcohol, we speculate that the aldehyde might undergo a Dakine type oxidation, thus producing a phenol (**VIII**). Condensation of **VIII** affords the heterocyclic compound **IX**, which after oxidation furnishes the precursor **X**. Selective formation of a five-membered ring instead of a six-membered ring might either be ascribed to the fact that formation of five-membered rings is kinetically favoured over the formation of six-membered rings or alternatively an enzyme might promote the selectivity of the reaction. Another route for the biosynthesis of **X** could proceed by the same pathway outlined for citrinin [33] with the crucial intermediate being 2,4-dihydroxy-5-methyl-6-(3-oxobutan-2-yl)isophthalaldehyde. A twofold Dakin type oxidation of this aromatic dialdehyde, followed by condensation might form **X**. Yet, as the twofold Dakin type oxidation would lead to a tetrahydroxylated aromatic compound, we deem this pathway less likely. Compound **X** finally undergoes a radical dimerization process as outlined in detail in Figure 4, with the sequence of oxidation and reduction reactions yielding the isolated compound **1**.

## 4. Materials and Methods

### 4.1. Sponge Collection

Ten sponges were collected from Hoga Island waters in the Wakatobi National Park, South East Sulawesi, Indonesia (5°28′24.68″S and 123°45′16.66″E) in August 2016 by scuba diving at 20 m depth (Figure 1), and documented as WK-P1, WK-P2, WK-P3, WK-P5, WK-P7, WK-P8, WK-P9, WK-P11, WK-P12, and WK-P20. According to reference [38], the specimens were categorized in the following genera: *Clathria* sp. WK-P1; *Suberea* sp. WK-P2; *Suberea* sp. WK-P9; *Neopetrosia* sp. WK-P3; *Cinachyrella* sp. WK-P5; *Carteriospongia* sp. WK-P8; *Hyrtios* sp. WK-P11; and *Aaptos* sp. WK-P12. Only 3–5 cm material were taken from each specimen. All samples were photo documented underwater and labelled before storage. Samples were placed in a zip-lock plastic bag, temporarily stored in a cool box and directly processed in the laboratory.

### 4.2. Fungal Isolation and Purification

The isolation of sponge-associated fungi was done according to Kjer’s protocol [39]. Sponges were initially sprayed with sterile natural seawater, cut into three pieces with an approximate size of 1 cm^2^ and placed into malt extract agar (MEA) (Himedia, Mumbai, India) medium (30 g malt extract, 5 g mycological peptone, 15 g agar, and 1000 mL sterile natural seawater, hereinafter referred to as marine MEA). The agar plates were incubated at room temperature (25 °C) for three days. The initial selection of fungal colonies was done based on phenotype, e.g., colony morphology and color [40]. In total, 20 fungal colonies were isolated and propagated until axenic cultures were obtained from ten sponges. In an initial screening, using the agar plug method, five strains were active and strain WK-P9 was selected for further investigation due to its prominent activity.

### 4.3. Molecular Identification of the Fungus

DNA amplification was performed using the Toyobo KOD FX Neo kit (Toyobo, Osaka, Japan). Fungus WK-P9 was grown in marine MEA for 3 days; a loop of mycelia was transferred into 2 µL PCR grade water, which served as template for colony PCR. The PCR mixture was set up in a total volume of 50 µL as follows: PCR grade water 9 µL, 2× PCR buffer for KOD FX Neo 25 µL, 2mM dNTPs 10 µL D, primer ITS1 (5’-tccgtaggtgaacctgcgg-3’, 10 pmol/µL) 1.5 µL, primer ITS4 (5’-tcctccgcttattgatatgc-3’, 10 pmol/µL) 1.5 µL, DNA template 2 µL (from mycelia), KOD FX Neo (1.0 U/µL) 1 µL. On a T100™ Thermal Cycler from Bio-Rad (Feldkirchen, Germany), the PCR program was run as follows: Denaturation initially at 95 °C for 3 min, followed by 35 cycles (denaturation at 95 °C for 3 min, annealing at 55 °C for 45 s, and extension at 72 °C for 1 min), then 72 °C extension for 7 min and cooling to 16 °C. The PCR product was Sanger-sequenced (1st BASE DNA sequencing services). Then, MEGA X was used to generate an alignment and the phylogenetic tree using the sequence data obtained. The phylogenetic tree was constructed using the Maximum likelihood and Neighbor-Joining analysis, with 1000 replications of bootstrap value (Figure S8). The sequence data was submitted to GenBank (Acc. Number: LC371661.1).

### 4.4. Isolation and Structure Elucidation

Three pieces of *P. citrinum* WK-P9 (each 1 × 1 cm^2^), which nearly covered the whole surface of a Petri dish, were inoculated onto malt extract broth (Himedia, Mumbai, India) media diluted by sterilized natural seawater (12 L) under clean-bench conditions, and cultivated for 12 days at 24 °C. The crude extract of *P. citrinum* WK-P9 (4.0 g) was collected after soaking the medium with ethyl acetate (EtOAc) using the ratio 1:3. The purification was initially performed by Silica (Wako, Japan) vacuum liquid chromatography (VLC) using a gradient composed of three different solvent systems-*n*-hexan:EtOAc (7:3); DCM:MeOH (85:15); EtOAc:MeOH (85:15), yielding five fractions. By activity-guided isolation, three active fractions were further purified by Sephadex LH-20 (GE Healthcare Europe GmbH, Freiburg, Germany), reverse phase silica C-18 (Interchim Puriflash 4125 Chromatography system with a Puriflash C18-HP 30μm F0080 Flash column), yielding 12 fractions. The final purification was conducted by HPLC (column EC 250/4.6 Nucleodur C18 Gravity-SB, 5μm) at a flowrate of 1 mL/min. The respective HPLC solvent gradients applied for the purification of the four compounds described in this paper are as follows: Compound **1** was obtained using a gradient of 55–60% MeOH/H_2_O within 25 min. Compound **2** was purified using a gradient of 40–46.5% Acetonitrile/H_2_O + 0.01% TFA within 15 min. Compound **3** was isolated using a gradient of 45–55% Acetonitrile/H_2_O + 0.01% TFA within 15 min. Compound **4** was obtained using isocratic elution of 87% MeOH/H_2_O + 0.01% TFA. 

Penicitrinone G (**1**): White amorphous solid; ${\left[\alpha \right]}_{D}^{24}$ = 16.25 (c 0.03, MeOH): ^1^H (600 HMz) and ^13^C (150 MHz) NMR (CD_3_OD), see Table 1; LC-HRESIMS *m/z* 385.1653 [M + H]^+^ and 407.1489 [M + Na]^+^ (calculated mass for C_22_H_25_O_6_: *m/z* = 385.1646). 

### 4.5. Antibacterial Susceptibility Tests

The antimicrobial activity of the crude extract and the following sub-fractions was conducted using the agar diffusion method [41]. The respective test bacteria (*Escherichia coli* K12, *Bacillus megaterium* DSM32, *Bacillus subtilis* JH642, *Micrococcus luteus* ATCC4698) were spread on Luria Bertani (LB) agar plates (10 g peptone, 5 g yeast extract, 5 g NaCl, 15 g agar, mixed with 1 L distilled water). For sample preparation, 15 µL of each crude extract (10 mg/mL dissolved in methanol) or fraction were added to a paper disk. Methanol was used as the negative control and carbenicillin (5 µL of a 50 mg/mL stock solution) (Carl Roth GmbH + Co., Karlsruhe, Germany) was used as positive control. The dried paper disks were subsequently positioned on the agar plate and incubated at 30 °C overnight. The diameter of the resulting inhibition zone was determined. 

Determination of the minimum inhibitory concentrations (MIC) of purified compounds **1**–**4** was carried out by micro broth dilution assays in 96 well plates. All compounds were dissolved in dimethyl sulfoxide (DMSO, Carl Roth GmbH + Co., Karlsruhe, Germany) and tested in triplicate. For *B. subtilis* DSM10 and *S. aureus* ATCC25923, an overnight culture (37 °C, 180 rpm) was diluted to 5 × 10^5^ cells/mL in cation adjusted Mueller Hinton II medium (Becton Dickinson, Sparks, NV, USA). *L. monocytogenes* DSM20600 was incubated for 2 days before the assay inoculum was adjusted using the same medium and growth conditions (Mueller Hinton II medium). As positive controls, dilution series of rifampicin, tetracycline and gentamycin (all Sigma Aldrich, St. Louis, MS, USA) were prepared (64–0.03 µg/mL). Cell suspensions without test sample or antibiotic control were used as negative controls. After incubation (18 h and 48 h for *L. monocytogenes*, 37 °C, 180 rpm, 80% rH) cell growth was assessed by turbidity measurement with a microplate spectrophotometer at 600 nm (LUMIstar® Omega BMG Labtech, Ortenberg, Germany).

The pre culture of *M. smegmatis* ATCC607 was incubated in Brain-Heart Infusion broth (Becton Dickinson) supplemented with Tween 80 (1.0% (*v*/*v*)) for 48 h at 37 °C and 180 rpm before the cell concentration was adjusted in cation adjusted Mueller Hinton II medium. Isoniazid (Sigma-Aldrich) was used instead of gentamycin as a third positive control. Cell viability was evaluated after 48 h (37 °C, 180 rpm, 80% rH) via ATP quantification (BacTiter-Glo™, Promega, Madison, WI, USA) according to the manufacturer’s instructions. 

*C. albicans* FH2173 was incubated for 48 h at 28 °C and 180 rpm before the pre culture was diluted to 1 × 106 cells/mL in cation adjusted Mueller Hinton II medium. For *A. flavus* ATCC9170, a previously prepared spore solution was used to prepare the assay inoculum of 1 × 105 spores/mL. Yeast and mold assays were incubated for 48 h at 37 °C, 180 rpm and 80% rH. For both, tebuconazole (Cayman Chemical Company, Ann Arbor, MI, USA), amphotericin B and nystatin (both Sigma Aldrich) were used as positive control (64–0.03 µg/mL). The readout was carried out by ATP quantification.

## 5. Conclusions

In summary, by applying bioassay-guided fractionation, four citrinin derivatives were obtained from the sponge-associated fungus *P. citrinum* WK-P9. Among those substances, the new derivative **1** was characterized for the first time, revealing a connection of monomers which has so far been unprecedented for citrinin derivatives. To provide an explanation for the formation of this new derivative, a biosynthetic hypothesis was proposed. Furthermore, all isolated compounds were screened for bioactivity. In this respect, penicitrinol J (**4**) showed moderate antimicrobial activity against *B. subtilis* JH642, *B. megaterium* DSM32, and *M. smegmatis* ATCC607. Even though *P. citrinum* is a well-investigated fungal species, such strains still have an inherent high possibility to deliver new specialized metabolites, as exemplified by the isolation of the new compound **1**. New strains in combination with variation in culture conditions, e.g., using the one strain many compounds (OSMAC) approach, thus represent a promising bioresource for natural product discovery. 

## Figures and Tables

**Table 1:** ^1^H (600 MHz) and ^13^C (150 MHz) NMR data of **1** (CD_3_OD; δ in ppm).

Position	^13^C		^1^H	HMBC
**1**	89.2	(CH)	4.43 qd (6.4, 3.6)	C-3, C-8, C-9
2	45.3	(CH)	2.99 qd (6.7, 3.6)	C-10, C-3, C-8
3	140.0	(C_q_)		
4	55.6	(C_q_)		
5	199.5	(C_q_)		
6	73.1	(CH)	3.63 s	C-4, C-5, C-7, C-8, C-3′, C-4′, C-5′, C-11′
7	184.7	(C_q_)		
8	150.3	(C_q_)		
9	20.6	(CH_3_)	1.24 d (6.4)	C-1, C-2
10	20.3	(CH_3_)	1.22 d (6.7)	C-1, C-2, C-3
11	16.1	(CH_3_)	1.325 s	C-3, C-4, C-5, C-6′, C-8
1′	88.3	(CH)	4.37 p (6.4)	C-3′, C-8′, C-10′
2′	47.1	(CH)	2.91 p (7.0)	C-1′, C-3′, C-8′, C-9′
3′	139.2	(C_q_)		
4′	55.1	(C_q_)		
5′	199.0	(C_q_)		
6′	71.9	(CH)	3.71 s	C-3, C-4, C-5, C-11, C-4′, C-5′, C-7′, C-8′
7′	184.2	(C_q_)		
8′	150.8	(C_q_)		
9′	20.7	(CH_3_)	1.30 d (6.4)	C-1′, C-2′
10′	19.3	(CH_3_)	1.36 d (7.0)	C-1′, C-2′, C-3′
11′	14.5	(CH_3_)	1.332 s	C-6, C-3′, C-4′, C-5′, C-8′

**Table 2:** Bioactivity of compounds **1**–**4**.

Microorganisms Tested	MIC (µg/mL) ^a^			
	Penicitrinone G (1)	Penicitrinone A (2)	Penicitrinone E (3)	Penicitrinol J (4)
*Bacillus megaterium* DSM32	>64	>64	>64	**16**
*Bacillus subtilis* JH642	>64	>64	>64	**16**
*Bacillus subtilis* DSM10	>64	>64	>64	**64**
*Micrococcus luteus* ATCC4698	>64	>64	>64	>64
*Mycobacterium smegmatis* ATCC607	>64	**32**	>64	**32**
*Listeria monocytogenes* DSM20600	>64	>64	>64	>64
*Staphylococcus aureus* ATCC25923	>64	>64	>64	**64**
*Escherichia coli* K12	>64	>64	>64	>64
*Candida albicans* FH2173	>64	>64	>64	>64
*Aspergillus flavus* ATCC9170	>64	>64	>64	>64

^a^ Activities are highlighted in bold.

## Supplementary Materials

The following are available online at https://www.mdpi.com/1660-3397/18/4/227/s1, Figures S1–S6: NMR data of compound **1**; Figure S7: LC-HRESIMS of compound **1**; Figure S8 phylogenetic tree of fungus *Penicillium citrinum*.
