# Supplementary material for: A New Citrinin Derivative from the Indonesian Marine Sponge-Associated Fungus Penicillium citrinum
Source: Mar Drugs. 2020 Apr 24;18(4):227. doi: 10.3390/md18040227 (PMC7230553; doi:10.3390/md18040227)

Supplementary Information

A New Citrinin Derivative from the Indonesian Marine Sponge-Associated Fungus *Penicillium citrinum*

Aninditia Sabdaningsih [1,2,3,5,6,*], Yang Liu [3,4,*], Ute Mettal [3,4], John Heep [4], Riyanti [3,4,10], Lei Wang [3,4], Olvi Cristianawati [1,5,6], Handung Nuryadi [5,7], Mada Triandala Sibero [5,6,9], Michael Marner [4], Ocky Karna Radjasa [5,8,9], Agus Sabdono [5,9], Agus Trianto [6,9] and Till F. Schäberle [3,4,11,*]

[1] Department of Coastal Resources Management, Faculty of Fisheries and Marine Sciences, Diponegoro University, 50275 Semarang, Indonesia; olvi.cristiana@yahoo.com (O.C);

[2] Department of Aquatic Resources, Faculty of Fisheries and Marine Sciences, Diponegoro University, 50275 Semarang, Indonesia

[3] Institute for Insect Biotechnology, Justus-Liebig-University of Giessen, 35392 Giessen, Germany; Ute.Mettal@chemie.uni-giessen.de (U.M.); riyanti@bio.uni-giessen.de (R.); Lei.Wang@agrar.uni-giessen.de (L.W.); till.f.schaeberle@agrar.uni-giessen.de (T.F.S.)

[4] Department of Bioresources of the Fraunhofer Institute for Molecular Biology and Applied Ecology (IME), 35392 Giessen, Germany; John.Heep@ime.fraunhofer.de (J.H.); Michael.Marner@ime.fraunhofer.de (M.M.)

[5] Tropical Marine Biotechnology Laboratory, Diponegoro University, Semarang 50275, Indonesia; handung.nuryadi87@gmail.com (H.N.); madatriandala@hotmail.com (M.T.S); ocky_radjasa@yahoo.com (O.K.R.); agus_sabdono@yahoo.com (A.S.);

[6] Marine Natural Product Laboratory, Diponegoro University, 50275 Semarang, Indonesia; agustrianto.undip@gmail.com (A.T.)

[7] Graduate School of Engineering and Science, University of the Ryukyus, 1 Senbaru, Nihihara, 903-0213 Okinawa, Japan; handung.nuryadi87@gmail.com (H.N.)

[8] Ministry of Research and Technology of the Republic of Indonesia, 10340 Jakarta, Indonesia; ocky_radjasa@yahoo.com (O.K.R.)

[9] Department of Marine Sciences, Faculty of Fisheries and Marine Sciences, Diponegoro University, 50275 Semarang, Indonesia; madatriandala@hotmail.com (M.T.S)

[10] Faculty of Fisheries and Marine Science, Jenderal Soedirman University, 53122 Purwokerto, Indonesia; riyanti.anti@gmail.com (R.)

[11] German Center for Infection Research (DZIF), Partner Site Giessen-Marburg-Langen, Giessen, Germany

* Correspondence: aninditiasabdaningsih@live.undip.ac.id (A.S.); Liu.Yang@agrar.uni-giessen.de (Y.L.); till.f.schaeberle@agrar.uni-giessen.de (T.F.S.); Tel.: +49-641-99-37140 (T.F.S)

Contents

Figure S1. ^{1}H NMR spectrum of compound **1** (600 MHz, CD_3OD) 3

Figure S1.1 Expanded ^{1}H NMR spectrum of compound **1** (600 MHz, CD_3OD) 4

Figure S2. ^{13}C NMR spectrum of compound **1** (150 MHz, CD_3OD) 5

Figure S3. COSY spectrum of compound **1** 6

Figure S4. HSQC spectrum of compound **1** 7

Figure S5. HMBC spectrum of compound **1** 8

Figure S6. NOESY spectrum of compound **1** 9

Figure S7. LC-HRESIMS of compound **1** 10

Figure S8 phylogenetic tree of fungus *Penicillium citrinum* 11

Figure S1 ^1H NMR spectrum of compound **1** (600MHz, CD_3OD)

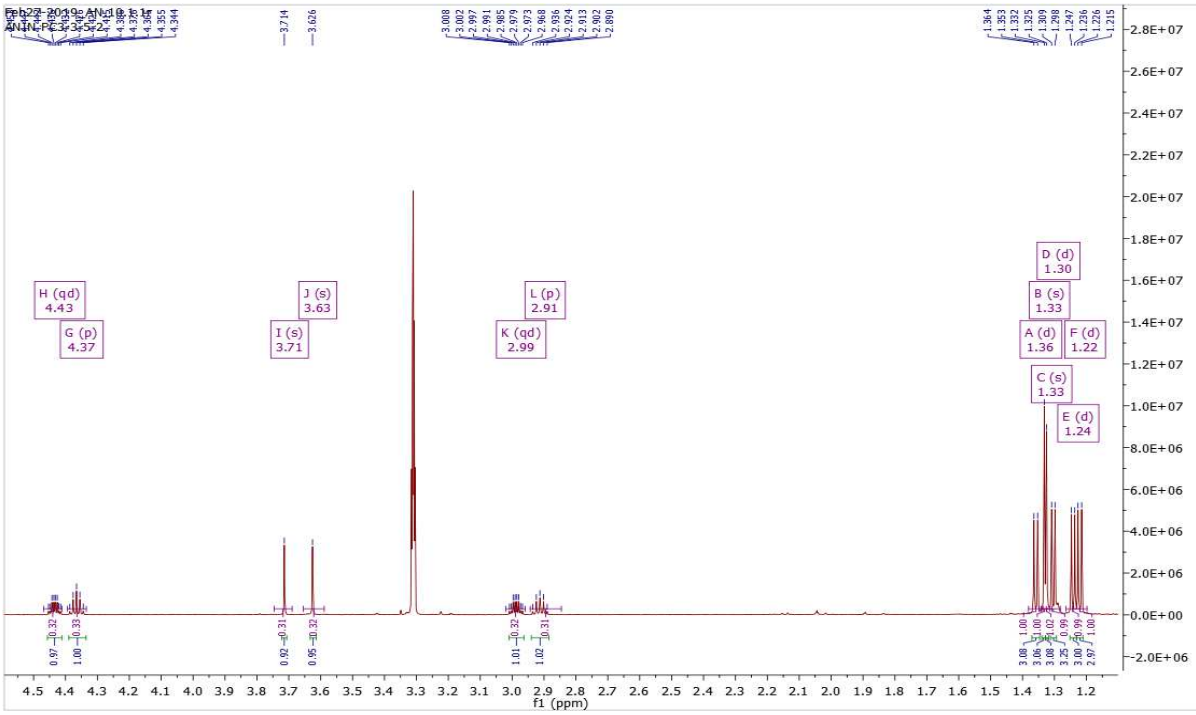

Figure S1.1 Expanded ^{1}H NMR spectrum of compound **1** (600 MHz, CD_3OD)

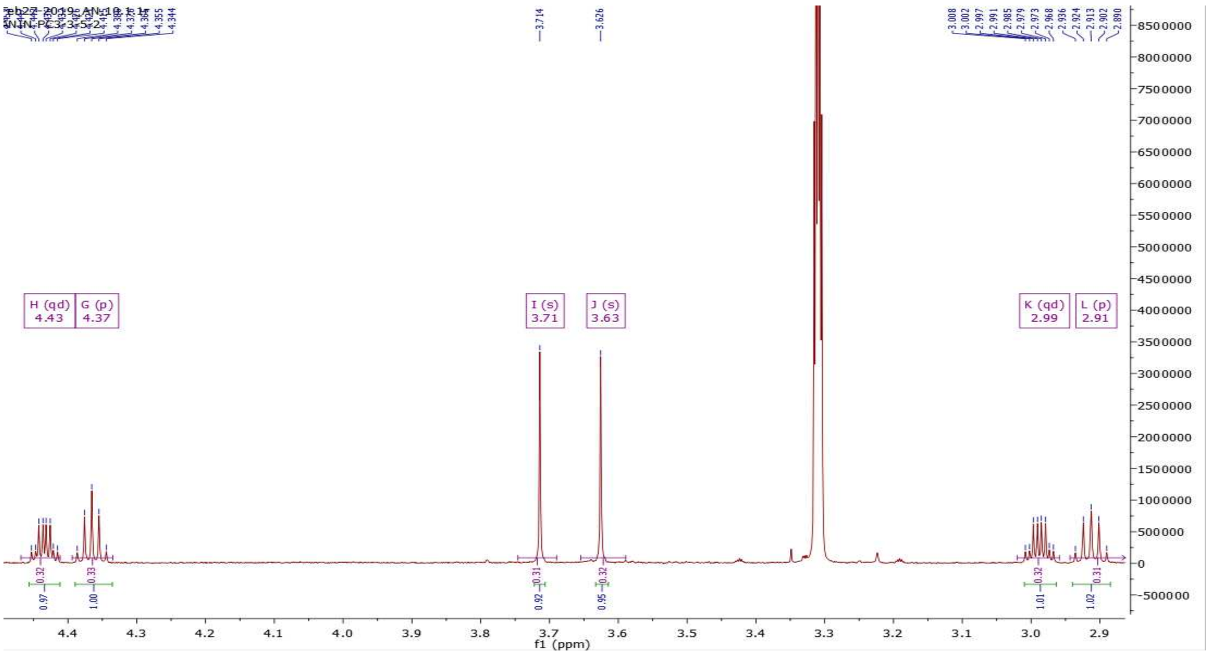

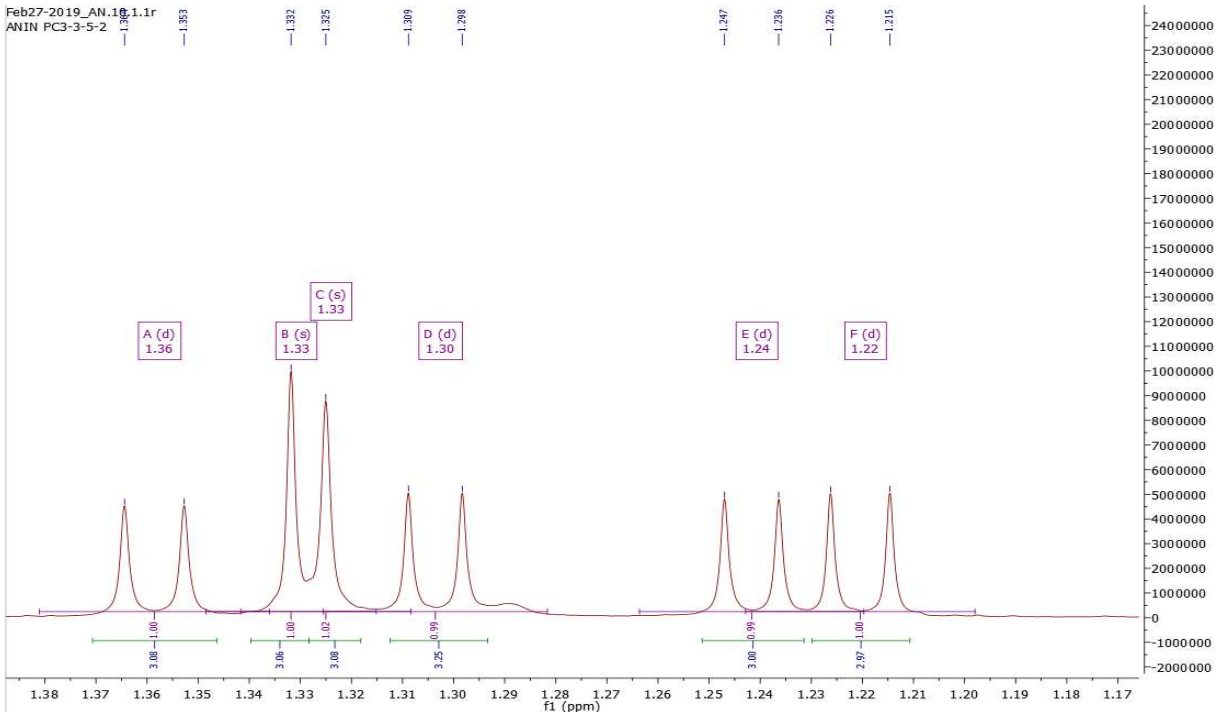

Figure S2 ^{13}C NMR spectrum of compound **1** (150MHz, CD_3OD)

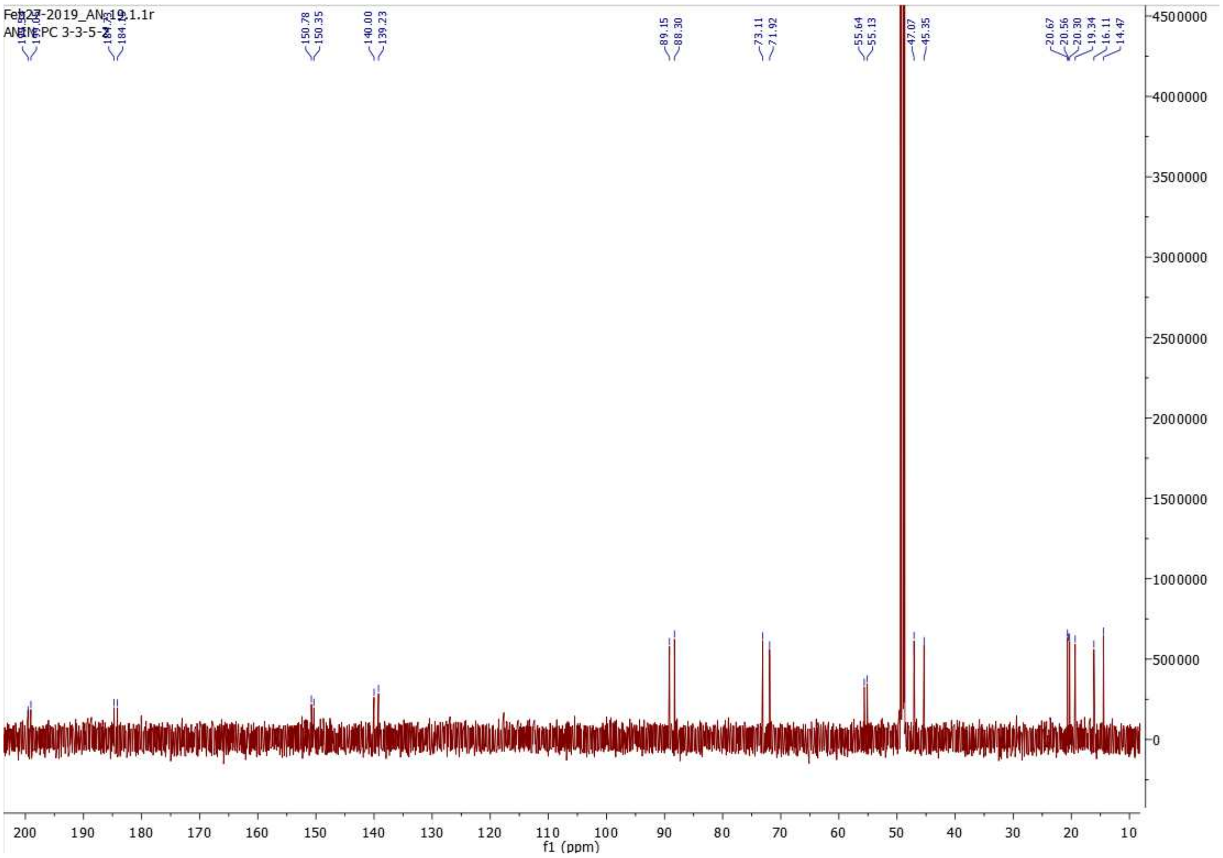

Figure S3. COSY spectrum of compound **1**

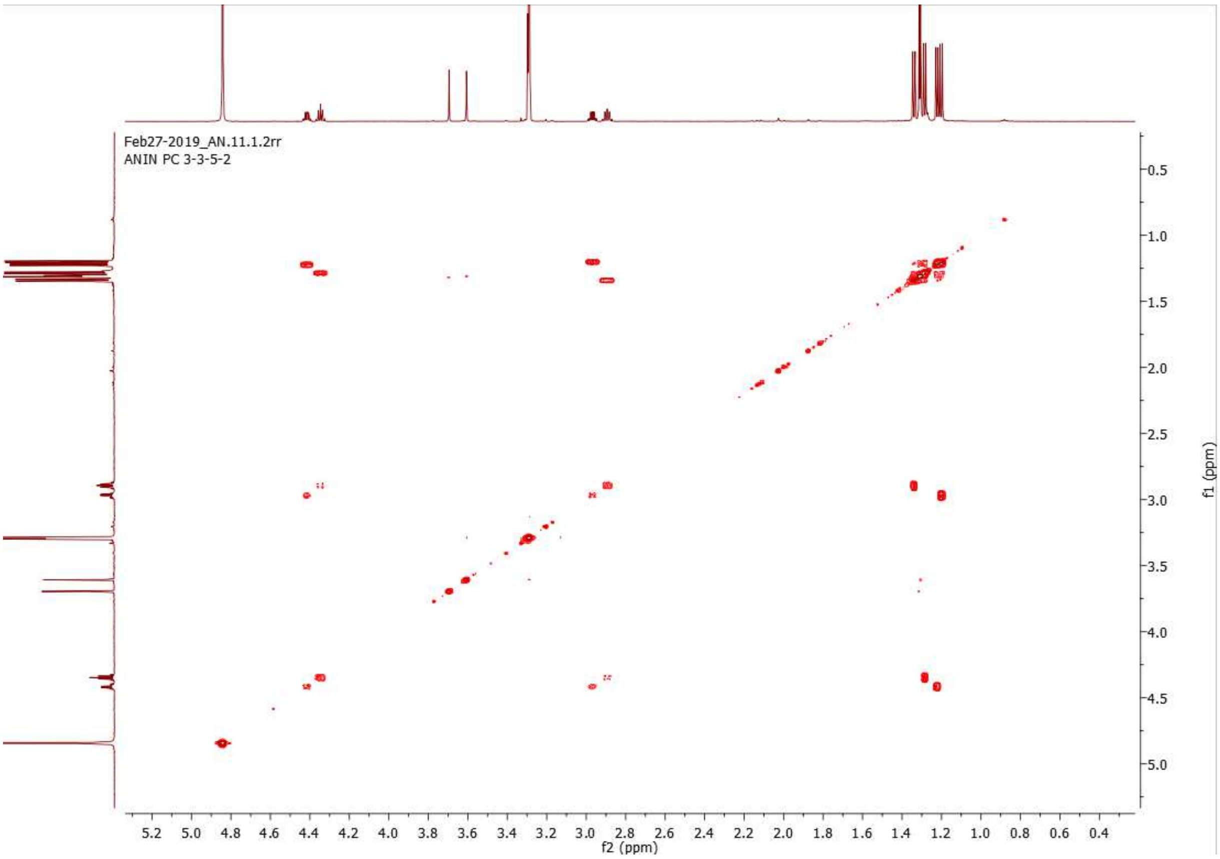

Figure S4. HSQC spectrum of compound **1**

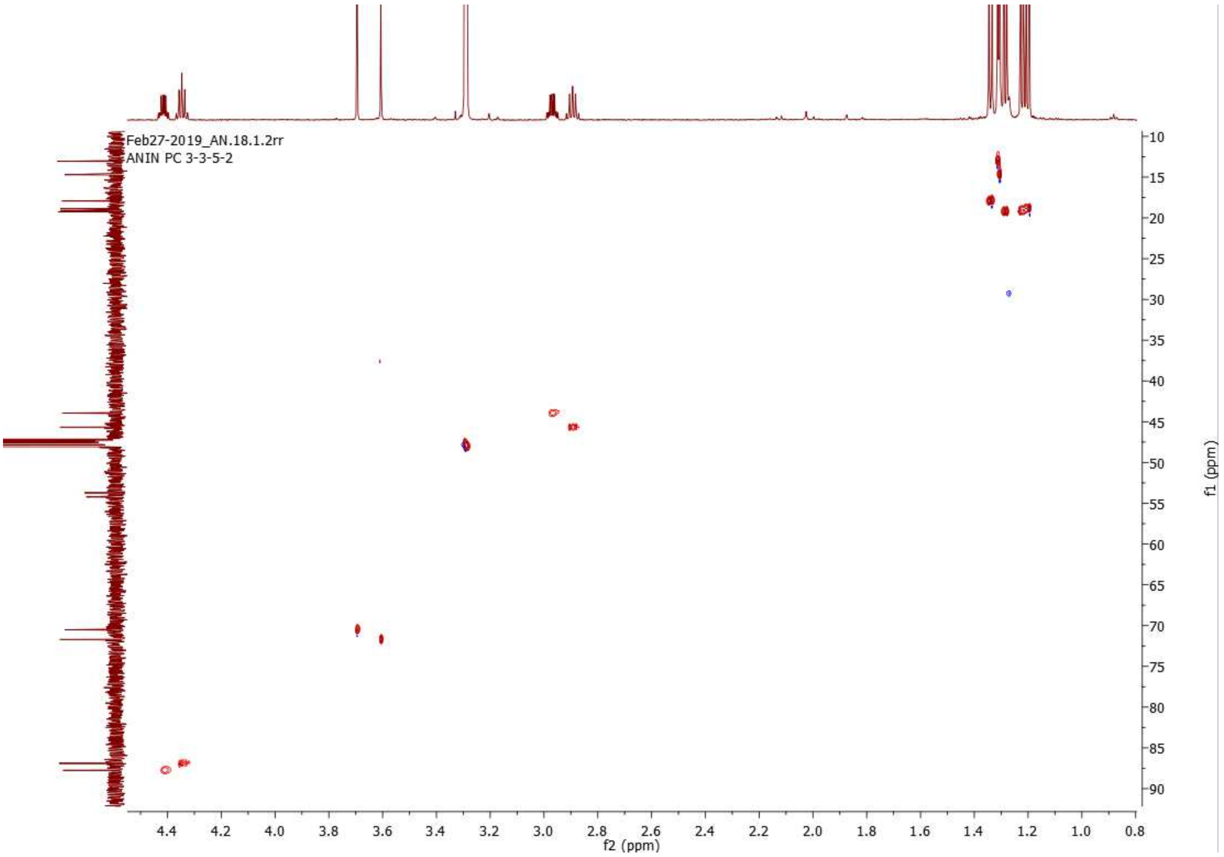

Figure S5. HMBC spectrum of compound **1**

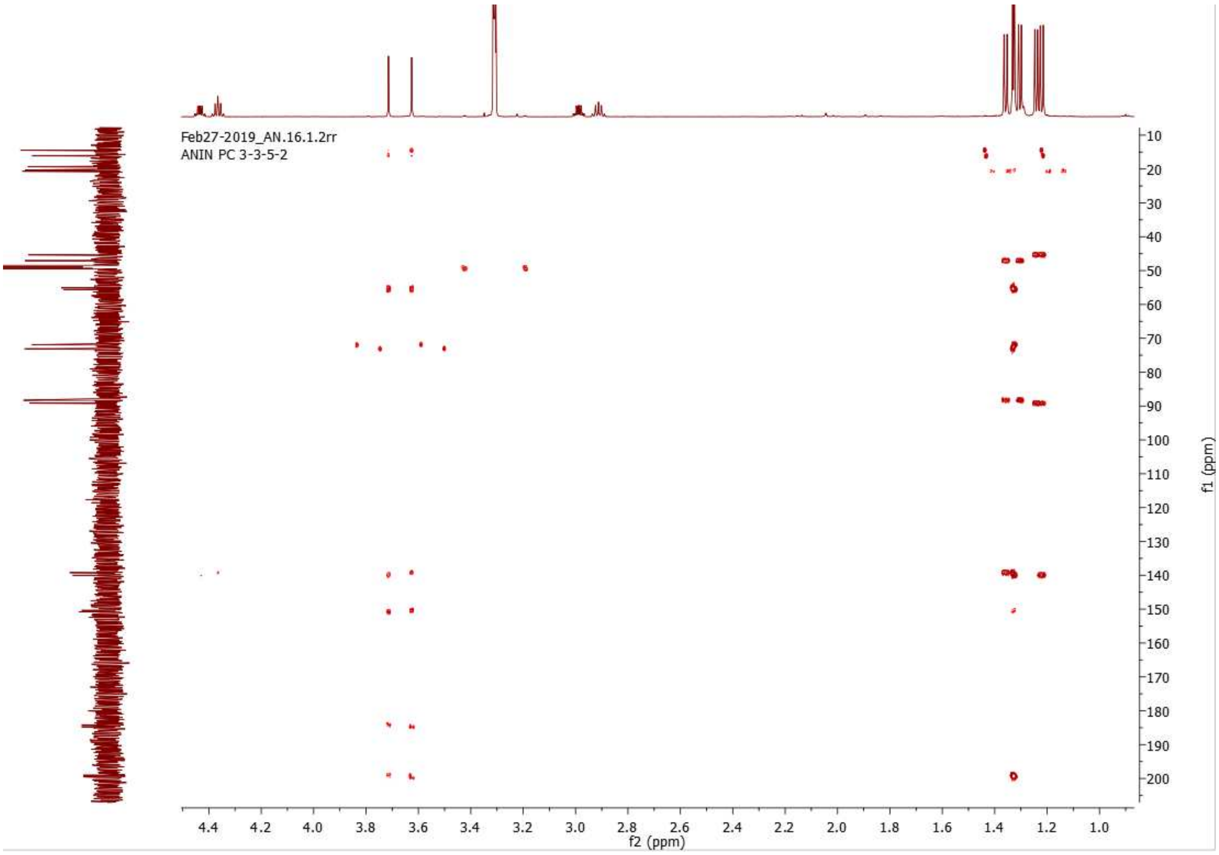

Figure S6. NOESY spectrum of compound **1**

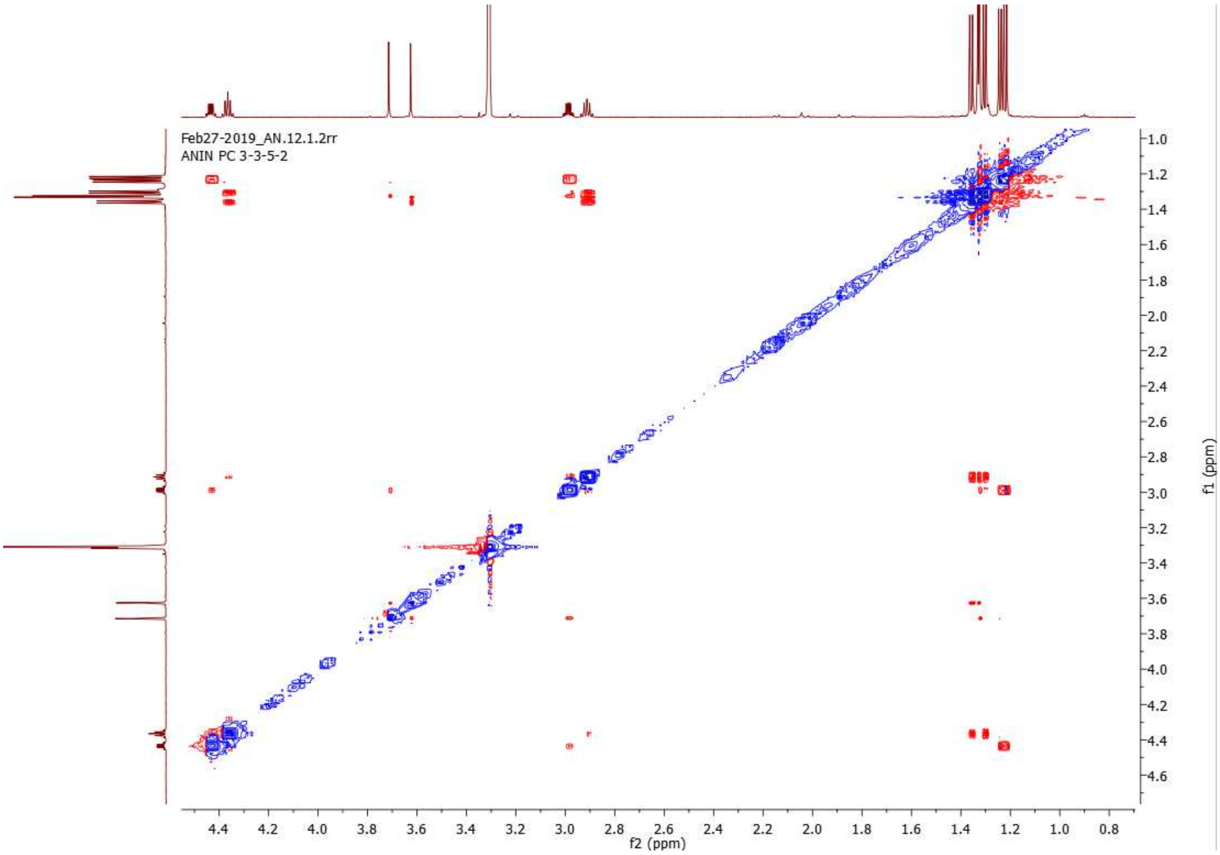

Figure S7. LC-HRESIMS of compound **1**

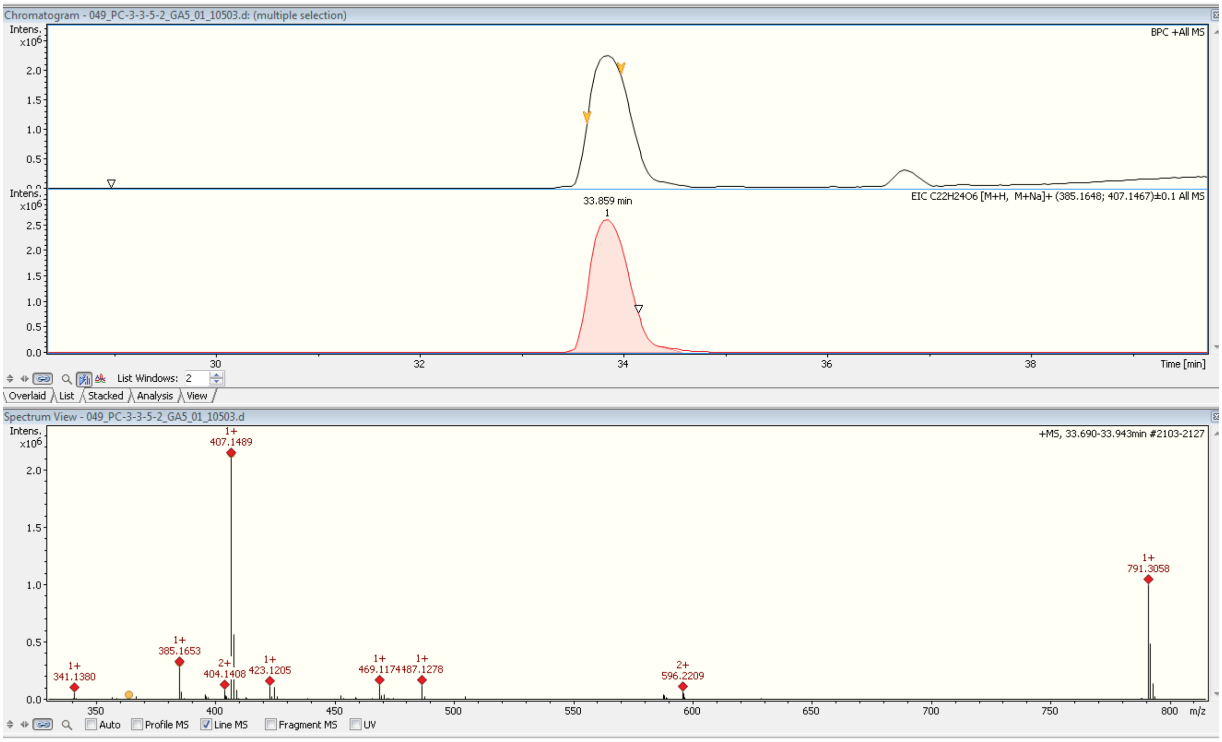

Meas. m/z	#	Ion Formula	m/z	err [ppm]	mSigma	# mSigma	Score	rd
385.1653	1	C22H25O6	385.1646	-1.9	4.7	1	100.00	10.
385.1653	2	C19H17N10	385.1632	-5.4	10.1	2	38.07	16.
385.1653	3	C23H21N4O2	385.1659	1.6	10.4	3	94.82	15.
385.1653	4	C18H21N6O4	385.1619	-8.8	18.7	4	9.46	11.

Meas. m/z	#	Ion Formula	m/z	err [ppm]	mSigma	# mSigma	Score
407.1489	1	C23H20N4NaO2	407.1478	-2.5	3.6	1	100.00
407.1489	2	C22H24NaO6	407.1465	-5.8	16.5	2	30.60
407.1489	3	C19H16N10Na	407.1452	-9.1	17.7	3	7.97
407.1489	4	C28H20N2Na	407.1519	7.3	23.8	4	15.03

Figure S8 phylogenetic tree of fungus *Penicillium citrinum*

A Maximum likelihood phylogenetic tree based on Internal Transcribed Spacer (ITS) region, the number of bootstrap replications 1000 was used to construct phylogenetic tree. The sponge-associated fungi was presented in red letters.

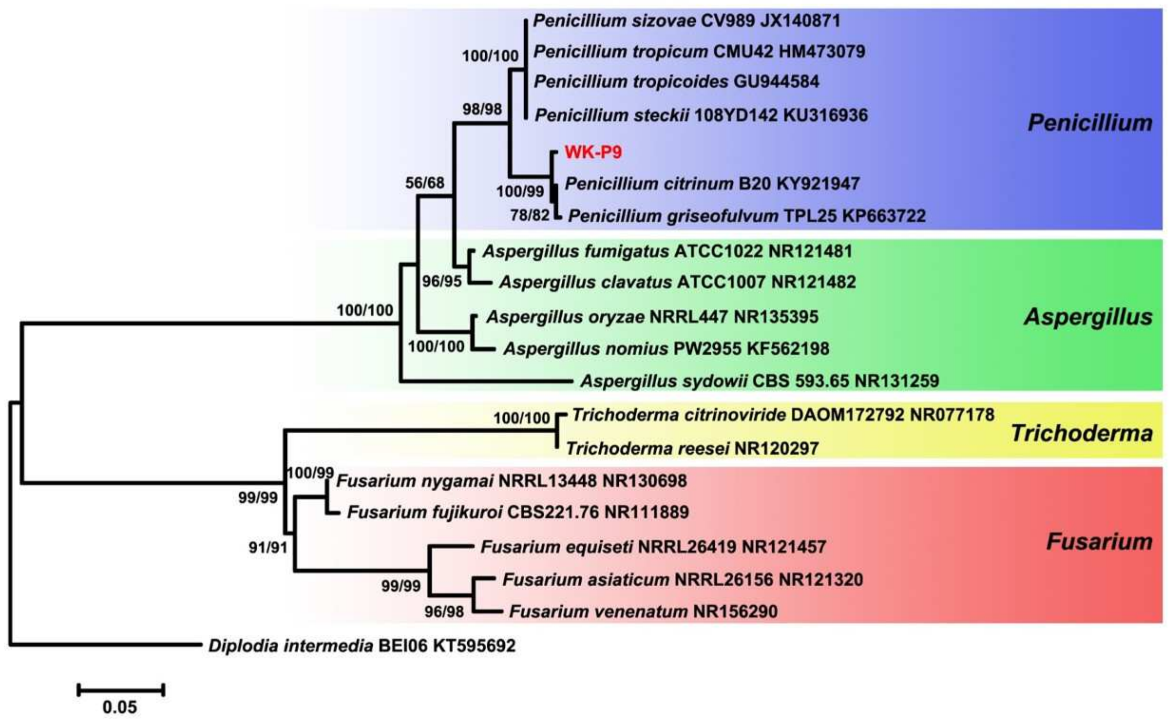

Supplement: Supplementary file 1 [file marinedrugs-18-00227-s001.pdf]
